# 

**Published:** 1902-06-07

**Authors:** 


### IMPORTANT NOTICE.

**A Double Number of "The Hospital" will be published next week (June 14) containing two Excellent Photogravure Plates of the Prince and Princess of Wales. The price of this Number will be 4d. SUBSCRIBERS desiring to obtain these Plates must send two penny stamps *at once***

**To the Manager, "The Hospital,"**
**28 & 29 Southampton Street,**
**Strand, London,**

**as otherwise they will not be issued with Subscription Copies.**

### NOTICE TO CORRESPONDENTS.

All MSS., Letters, Books for Review, and other matters intended for the Editor should be addressed The Editor, "The Hospital," The Hospital Building, 28 & 29 Southampton Street, Strand, W.C.

The Editor cannot undertake to return rejected MS., even when accompanied by stamped directed envelope.

### Notice to Advertisers and Subscribers.

All Advertisements, Orders for Copies of The Hospital, and Business Communications should be addressed to **THE MANAGER, (Not to the Editor)**, The Hospital, 28 & 29 Southampton Street, Strand, London, W.C.

The Cost of Subscription, post free, is as follows.—Per week, 2½d.; per quarter, 2s. 8d.; per half-year, 5s. 3d.; per annum, 10s. 6d. Foreign:—Per annum, 15s. 2d., payable, in all cases, in advance.

### BOOKS RECEIVED.

Baillière, Tindall and Cox.

"Clinical Lectures on Stricture of the Urethra and Enlargement of the Prostate." By P. J. Freyer, M.A., M.D.

"The Hair and its Diseases." By David Walsh, M.D.Edin.

"Practitioner's Handbook of Diseases of the Ear and Naso-Pharynx." By H. Macnaughton-Jones, M.D., W. R. H. Stewart, F.R.C.S.Ed., William Milligan, M.D, Herbert Tilley, M.D., Ambrose Birmingham, M.D., and Robert Dwyer Joyce, F.R.C.S.I.

Publishing.

"Holidays in Eastern Counties." Edited by Percy Lindley.

Thomas Burleigh.

"Science in the Daily Meal." By Albert Broadbent, F.R.H.S.

Agricultural and Horticultural Association.

"One and All Gardening, 1902." Edited by E. O. Greening.

Harrison and Sons.

"The Rational Cure of Obesity." By William Banting.

C. M. Löhr.

"The Nurse's Report Book." By C. M. Löhr.

Spottiswoode and Co.

"Minutes of the General Medical Council."

Central Stationery and Printing Company, Liverpool.

"Don'ts. A Short Address to Nurses." By Emily F. Stewart Brown.

John Wright and Co.

"Protoplasm: its Origin, Varieties, and Functions." By John W. Hayward, M.D.

Edward Arnold.

"A Text-book of Physics." By R. A. Lehfeldt, D.Sc.

William Reeves.

"Facts about Flogging." By Joseph Collinson.

Rebman, Limited.

"Tuberculosis as a Disease of the Masses." By S. A. Knopf, M.D.

### The Student of Medicine.

### APPOINTMENTS.

**J. F. E. Bridger**, M.R.C.S., L.R.C.P.Lond., appointed Civil Surgeon to the troops and Boer prisoners stationed at Antigua. **Major E. Harold Brown**, M.D., I.M.S., appointed Clinical Assistant to the Chelsea Hospital for Women. **Clayton W. H. Greene**, B.A., M.B., B.C.Cantab., F.R.C.S.Eng., appointed Surgical Registrar to St. Mary's Hospital, Paddington. **Norman E. Harding**, M.B., Ch.B.Edin., appointed locum Assistant Medical Officer to the Durham County Asylum. **Owen C. Jones**, M.B.Oxon., M.R.C.S.Eng., appointed Certifying Factory Surgeon for the Ilfracombe District of Devonshire. **H. W. Martindale Kendall**, M.R.C.S.Eng., appointed Ophthalmic Surgeon to the Wellington Hospital, N.Z. **F. C. Leonard**, M.D., appointed Clinical Assistant to the Chelsea Hospital for Women. **V. Warren Low**, M.D., B.S., F.R.C.S., appointed Surgeon to the Out-patients, St. Mary's Hospital. **Ernest Arthur Milner**, M.B., C.M.Edin., appointed Medical Officer of Health for Lostwithiel. **Geo. Potts**, L.R.C.P. and S., appointed House Surgeon, Kent County Ophthalmic Hospital, Maidstone. **F. Lionel Provis**, M.R.C.S., L.R.C.P., appointed Registrar to the Chelsea Hospital for Women. **John Geddes Scott**, M.R.C.S., L.R.C.P.Lond., appointed Medical Officer to the Western Telegraph Company, Madeira. **E. Taunton**, M.R.C.S.Eng., L.R.C.P.Lond., appointed Assistant Medical Officer to the St. Pancras Workhouse. **John Mearns Taylor**, M.B., C.M.Edin., appointed Medical Officer and Public Vaccinator to Skelmorlie District, Largs Parish; also Medical Officer and Public Vaccinator to the Inverness District, Inverness Parish Council.

St. Thomas's Hospital.—New house appointments:—House Physicians—**H. W. Sinclair**, M.B.Lond., L.R.C.P., M.R.C.S.; **K. E. Crompton**, B.A., M.B., B.C.Cantab. Assistant House Physicians—**A. D. Hamilton**, M.B.Lond., L.R.C.P., M.R.C.S.; **C. H. Sedgwick**, B.A., B.C.Cantab. Obstetric House Physicians—(*Senior*) **T. D. Miller**, L.R.C.P., M.R.C.S.; (*Junior*) **R. E. Roberts**, M.B., B.Sc.Lond.

28 & 29 SOUTHAMPTON STREET, STRAND, W.C.

